# Evaluating and supporting leadership, management, and mentoring: a framework for catalyzing responsible research and healthy research environments

**DOI:** 10.3389/frma.2025.1569524

**Published:** 2025-07-01

**Authors:** Tristan McIntosh, Alison L. Antes

**Affiliations:** Department of Medicine, Bioethics Research Center, Washington University School of Medicine, St. Louis, MO, United States

**Keywords:** leadership, management, mentoring, research teams, scientific excellence, evaluation

## Abstract

Those who lead research teams have myriad roles and responsibilities that are pivotal to both producing rigorous and responsible scientific work and creating a supportive research environment that cultivates this work. We begin by presenting a leadership, management, and mentoring (LMM) framework focused on three critical roles researchers must play that have direct impact on the scientific work, the work environment, and research team dynamics: the role of research leader, research manager, and research mentor. Research leadership involves fostering a healthy research culture by building relationships where team members feel respected and supported. Research management involves providing oversight and direction of day-to-day operations to ensure tasks are done effectively, rigorously, and responsibly. Research mentoring involves providing opportunities and support to team members so that they develop professionally and build their careers. While these three roles are distinct, there is overlap in the professional, interpersonal, and intrapersonal skills that underlie their effective performance, such as communication, active listening, emotion management, and self-reflection. We also draw attention to some of the challenges when performing LMM roles. A variety of sources and types of evaluation measures may be used to comprehensively assess the functioning of a research team and its leader(s). We illustrate key domains for measurement, example indicators of effectiveness in those domains, and examples of the types of measures that could be used for evaluation. We discuss how top-down evaluation, bottom-up evaluation, and self-evaluation methods could be employed for data collection and note that each of these methods has strengths and limitations. We recommend multiple sources and types of data but acknowledge that evaluation must be feasible and practical. We note best practices and key implementation considerations for each method of measurement. When combined, these three methods provide a robust approach for evaluating LMM. We conclude with a description of key considerations for supporting the evaluation and application of LMM in real-world settings at academic institutions. Such considerations include senior leadership buy-in and communication about LMM expectations and providing appropriate framing, time, support, and incentives for LMM. We also highlight institutional risk factors that may inadvertently undermine LMM goals.

## Introduction

Those who lead research teams have myriad supervisory roles and responsibilities that are pivotal to conducting rigorous, responsible scientific work *and* creating a healthy research environment. The importance of leadership, management, and mentoring (LMM) in the scientific enterprise has garnered greater awareness in the last decade or so (Heemstra and Garg, 2022; Shuler et al., 2021; Pizzolato et al., 2022; Kvaskoff and McKay, 2014; Brookes et al., 2017; Antes et al., 2016). Yet, actively evaluating and supporting LMM is not the norm in research institutions. We propose it should become a priority. An essential step is to gain clarity about what LMM are in the scientific research setting. The purpose of this paper is to define three specific roles—research leader, research manager, and research mentor—and to elucidate the tasks and responsibilities of these distinct, yet related roles. We illustrate behaviors, skills, and practices necessary to perform these roles well, discuss how metrics of effective LMM can be evaluated, and how academic institutions can support this process. Before we explore LMM and its evaluation, we discuss what makes LMM necessary in scientific settings, why LMM matters for science, and how LMM benefits academic institutions.

### What makes LMM necessary in science

Science seeks to expand knowledge, solve societal problems, and improve public wellbeing. To this end, researchers are responsible for upholding professional and ethical standards and maintaining public trust (DuBois and Antes, 2018). A unique aspect of the research enterprise is training the next generation of scientists to uphold these standards through mentoring, and the shift from the “lone scientist” to a team approach for tackling complex research questions (Adams, 2014; National Academies of Sciences Engineering and Medicine, 2019). Multidisciplinary teams with diverse expertise and roles are common, especially in fields aiming to translate findings into innovation that benefit society (Hall et al., 2018). The composition of these collaborative teams may change frequently, especially with the entry and exit of students and research trainees, making LMM essential and adding to its complexity.

Leaders in research teams often juggle multiple roles, such as mentoring students, supervising lab members, maintaining a healthy work environment, securing funding, and complying with regulations. The way researchers lead, manage, and mentor their teams directly affects the development of future scientists and the ethical conduct of research. Moreover, there are multiple ways to lead, manage, and mentor effectively, as there is no single approach to LMM that is universally effective across all contexts. Effective LMM is critical for planning long-term research agendas, navigating uncertainties, and ensuring high-quality, impactful, and credible research. A positive team environment, where members feel engaged, safe, and supported, fosters diversity, upholds integrity in research, and reduces the risk of research noncompliance or misconduct (Antes et al., 2019a,b, 2024; McIntosh et al., 2023).

### Why evaluating LMM matters

There is debate about the origins of the phrase “what gets measured gets managed,” but it serves as a guidepost for fostering LMM in institutions and teams. This statement suggests that leaders should measure what they want to prioritize by identifying and tracking relevant indicators over time. The value of this statement lies in its emphasis on the importance of evaluation. It is not enough to hope or assume effective LMM will happen; data-driven insights are what will add value in various ways. LMM metrics can inform professional development needs of research leaders, offering constructive feedback on LMM and team strengths and areas for growth. LMM metrics can come from a range of sources, such as from a researcher's department chair or someone in a similar role, members of a researcher's team (e.g., research assistants, staff), and self-evaluation and reflection exercises. Feedback is particularly valuable when it provides insights into team members' perceptions and experiences, highlighting areas of alignment or misalignment with the leader's views. This can help leaders make incremental changes and track progress over time. Effective collection of and action on team feedback supports team voices, gauges job satisfaction, motivation, and engagement, and improves these metrics when necessary.

As with any evaluation effort, caution should be exercised to avoid unintended consequences. Consider Goodhart's Law, which suggests that when a given measure becomes the primary focus, its effectiveness becomes limited if the goal of using that measure becomes too rigid or narrow (Elton, 2004). For example, if researchers are rewarded solely on the number of publications, the research quality and team environment may be neglected and suffer as a result. The potential negative downstream consequences of focusing on only one measure of performance is why evaluating LMM using a diverse range of metrics, and from different sources (e.g., superiors, subordinates, peers, and self) is so important. A well-rounded battery of assessments can help avoid the limitations of one LMM indicator alone. Understanding the impact of using certain measures of LMM can help champion effective and appropriate use of these measures. Goodhart's Law also reinforces the importance of having valid LMM measures that actually measure what they intend to.

### How academic institutions benefit from supporting evaluation and implementation of LMM

Broadly, measurement helps institutional leaders determine if behaviors within research teams align with the institution's stated priorities and values. Traditionally, researchers have had the independence to run their research programs as they see fit, with institutions mainly aware of their traditional academic outputs (e.g., papers, funding). Regular evaluation of LMM, however, fosters accountability and alignment with institutional goals. Moreover, effective evaluation and support for LMM promotes both the quality of work and wellbeing of individuals within research teams. Related to the broader scientific enterprise, excellent LMM aims for research excellence—characterized by productive, efficient, trustworthy, and credible research that prepares the next generation of researchers for future challenges. Supporting LMM fosters a healthy institutional culture, improves institutional reputation, attracts and retains talent, mitigates risk of research misconduct, and furthers the institutional mission. Additionally, LMM skills enhance adaptability during crises or change at the local, national, and global levels (Antes et al., 2023).

We draw on theories and research from the workplace psychology and leadership literature and studies on LMM in scientific research to present a conceptual framework for the roles of research leader, manager, and mentor. We provide indicators and example measures of LMM effectiveness and discuss three approaches to evaluating LMM. We conclude with practical considerations for supporting and implementing routine evaluation of LMM in research institutions.

## Research leadership, management, and mentoring

### Defining leadership, management, and mentoring (LMM)

Scholars have attempted to unearth what constitutes effective LMM across various domains. For instance, early leadership theories emphasized innate traits such as personality and intelligence that were necessary qualities of leaders (Liden et al., 2025; Judge et al., 2002; Yukl, 2013; Dinh et al., 2014). Later, scholars focused on behaviors leaders display, and leadership scholars then turned toward situational approaches where a leader must adapt their style to specific situations and the needs of their team members (Jago, 1982; Yukl et al., 2002). More contemporary approaches to leadership shifted to styles of leadership, such as transactional styles that use authority, rewards, and coercion to monitor performance, or transformational styles that motivate people through inspirational messages, intellectual stimulation, embodying values as a role model, and fostering trust and ownership (Judge and Piccolo, 2004; Young et al., 2021). Further, another contemporary shift has suggested that leadership can be shared among a group, leading to models of collective leadership (Benmira and Agboola, 2021).

Contemporary scholars who have aimed to understand the underlying cognitive and social skills of leaders, managers, and mentors offer particular value for those aiming to develop LMM capabilities and evaluate LMM (Mumford et al., 2000a,c, 2017; Riggio and Lee, 2007; Riggio and Reichard, 2008). Skills are learned abilities or the competence to perform specific tasks; that is, skills can be acquired through training and practice. Thus, a skills-based approach assists in identifying what needs to be learned to foster LMM effectiveness and the kinds of behaviors that leaders, managers, and mentors would be expected to competently perform. This work suggests that leaders, managers, and mentors need cognitive skills like creative thinking, planning, and idea evaluation, and social or interpersonal skills such as communication, active listening, conflict resolution, emotional intelligence, and fostering collaboration and positive team dynamics (Mumford et al., 2017, 2000b, 2007; Boyatzis et al., 2007, 2013; Castro et al., 2022; Chopin et al., 2012).

Technical scientific skills are also important for leadership, management, and mentoring, but they are not sufficient for LMM excellence. Historically in science, those who are technically competent have been elevated to roles in scientific leadership, management, and mentoring. However, technical skills are different than interpersonal and cognitive skills, which are required for effective LMM and warrant targeted development. In our proposed LMM framework, we focus specifically on what behaviors and practices you see a leader doing and what skills they need to engage in these behaviors because the behaviors and skills are observable practices that can be trained, developed, measured, and evaluated.

Given the complex evolution of LMM theories, we propose the following definitions of each role. A research leader sets the vision and strategy for their research program, secures needed resources, builds partnerships, and engages stakeholders to drive research inquiry and innovation. In their teams, they must foster a productive, collaborative, and ethical research culture by building relationships and developing a team environment where people feel respected, valued, and supported (McIntosh et al., 2020a; Kotter, 1990, 2008; Toor and Ofori, 2008). A research manager oversees and directs the day-to-day operational aspects of research projects and the work processes of team members to ensure tasks are executed effectively, rigorously, and responsibly (Antes et al., 2016; Simonet and Tett, 2013). A research mentor provides opportunities and supports team members to learn research skills, develop and grow personally and professionally, and build their careers (National Academies of Sciences Engineering and Medicine, 2019; Cho et al., 2011; Alegria et al., 2019).

These roles may be carried out by one individual, such as the principal investigator, or they could be carried out by more than one individual in a research team or laboratory. For instance, lab managers, senior graduate students, and postdoctoral researchers often take on supervisory and mentoring roles for less experienced team members. For the purposes of this paper, we focus on a primary source of LMM, such as a faculty member who serves as lab head or principal investigator, but we acknowledge there may be others sharing in providing support for some of these roles. A person does not need to have a formal role or title to engage in LMM. We further do not distinguish between types of team members, assuming team members could be any kind of research personnel, from undergraduate students to graduate students, postdoctoral researchers, or professional research staff. Indeed, effective LMM involves attunement to the individual needs, skills, and interests of people, particularly because people come from diverse backgrounds, perspectives, and disciplines (White-Lewis et al., 2022).

The roles of research leader, manager, and mentor are distinct yet overlapping. This perspective suggests that the domains of leadership, management, and mentoring could be conceptualized as a Venn diagram with overlapping circles, with each role having unique features but also sharing common features. Elucidating their unique facets acknowledges the complex functions of each role and their contributions to research effectiveness. Recognizing their overlap illustrates they are intersecting processes, each important to research excellence, and allows for identification of shared areas to target development that would improve the execution of each role.

Table 1 illustrates key areas of distinction between LMM. Leadership scholars typically describe leadership and management as distinct, yet complementary, noting that both are needed for organizational functioning and success (Kotter, 1990; Toor and Ofori, 2008; Simonet and Tett, 2013). Leaders set the strategic direction, create and motivate teams, build relationships, and inspire trust in people, while managers create systems and processes to produce reliable work, solve emerging problems, and coordinate tasks. Leaders focus on “what” and “why”, while managers focus on “how” and “when”. Mentors occupy a critical space in the scientific enterprise in that their focus is developing the next generation of scientists (Sambunjak et al., 2006; Feldman et al., 2010; Ragins and Kram, 2007; Brown et al., 2009). Mentors provide advice, guidance, and direction to individuals to develop their research skills to become independent researchers, and effective mentors meet their mentees' needs for personal development and psychosocial support (National Academies of Sciences Engineering and Medicine, 2019).

**Table 1:** Overview of LMM.

	**Research Leader**	**Research Manager**	**Research Mentor**
**Objective**	Build a team and create the conditions for success.	Organize and coordinate the team, the work, and resources.	Develop skills and provide guidance to others on their career paths.
**Primary focus**	Research vision, strategy, resources, people, and work environment.	Daily operations and team processes to complete projects.	Individual mentee or staff needs, goals, and progress.
**Primary responsibility**	Building quality relationships and healthy team interactions.	Fostering and overseeing effective procedures and work processes.	Advising, training, and guiding others.
**Signs of effectiveness**	Shared vision, good team dynamics, and positive work environment.	Problems are addressed, progress is made, and data are reliable and trustworthy.	Being a sought-out mentor, mentee independence, and mentee satisfaction.

Illustrating the unique features of each LMM role is useful, but the roles are intersecting and mutually supporting. For example, a manager is most effective if they are also a capable leader who can form quality relationships with their team. Excellent leaders are more effective when, like managers, they understand the nature and extent of coordination and resources necessary to execute their vision. Mentors are most effective not only when they develop individual people, but when they are also capable leaders who foster a work environment that is productive and developmental for everyone. It is often the degree to which a given role is enacted at a given time that matters most, not which specific role someone has (Simonet and Tett, 2013).

These overlapping roles collectively promote productivity, problem solving, goal attainment, learning and growth, team engagement, and persistence during challenges. Effective LMM ensures individuals understand their tasks, know how to perform them, and know where to seek help. Additionally, effective LMM helps people to feel valued through open communication, fair recognition, and shared credit for achievements. Research leaders, managers, and mentors do not need to be perfect; effectiveness lies in candid self-reflection and a mindset of continuous improvement (Antes and DuBois, 2018).

### Skills necessary for effective LMM

A crucial way in which these roles overlap is in the set of professional and social skills that underlie their effective performance (Mumford et al., 2000b; Simonet and Tett, 2013). To perform LMM roles well, strong interpersonal and intrapersonal skills are needed along with technical and scientific expertise (Antes and DuBois, 2018; Mumford et al., 2002; Day et al., 2014). Interpersonal skills, such as communicating in a manner that fosters good team dynamics and providing psychosocial support, are crucial for fostering creativity, innovation, and learning (Edmondson and Lei, 2014; Frazier et al., 2016; Robledo et al., 2012). These skills help create an emotionally and psychologically safe environment where team members can share ideas, take risks, and learn from failures (Delizonna, 2017). Effective leaders use empathy, active listening, and open communication to build trust and maintain positive relationships (Groysberg and Slind, 2012; Wefald, 2022; Kliewer, 2022). They also value and embrace differences among members of their team (Marshall et al., 2023; Bourke and Espedido, 2019).

Intrapersonal skills, including self-regulation, self-direction, and emotional management, enable leaders to recognize their strengths and limitations, manage their emotions, and adapt to challenges (Neck and Houghton, 2006; McIntosh et al., 2020b). Leaders with strong intrapersonal skills can respond constructively to mistakes and frustrations, fostering trust and positive morale within the team. Self-reflection is also vital, allowing leaders to assess their actions and improve continuously (Ashford and DeRue, 2012; Nesbit, 2012; Porter, 2017). A focus on both intra- and interpersonal skills enhances leaders' effectiveness, creating a supportive and adaptive research environment (Cohen and Cohen, 2018). While scientific training develops technical skills, LMM effectiveness relies on these human aspects of leadership. Developing both skillsets is essential to fostering a productive and resilient research community.

### Effectiveness in LMM roles

Many of the behaviors, practices, and outcomes of effective LMM could be conceptualized as outcomes and/or inputs through a self-reinforcing feedback loop. Positive behaviors and outcomes are mutually amplifying, creating a cycle of growth and success. For example, a leader fosters trust by being supportive and communicating openly. Team members then feel safe to share ideas, take risks, and they understand expectations. The team's collaborative efforts lead to better outcomes, reinforcing trust, openness, and the value of clear communication, which, in turn, leads to better collaboration, productivity, and success. The group builds a culture of continuous improvement and innovation, which drives additional success. This example illustrates how deliberate, consistent LMM can create cycles that build momentum and create long-term sustainable success.

In Tables 2–4, we outline broad domains of practices that contribute to and result from effective LMM, and provide sub-dimensions illustrating more specific behavioral indicators of the domains. These are not exhaustive lists of all LMM domains, but these domains offer a set of important signs of LMM effectiveness. In selecting domains, for leadership and mentoring, we focus particularly on those relying on interpersonal and intrapersonal skills, which often require greater awareness and development among scientists. For research management, we focus on administrative and operational aspects of the research group, including coordination of team members. We draw from existing research on leadership, management, and mentoring in the sciences where possible, and some organizational and leadership research outside of the scientific domain. It is also worth noting that the largest body of knowledge is on mentoring in the sciences, whereas more empirical research on leadership and management in science specifically is needed and would help advance the field. Our aim was to present essential sub-dimensions that are relatively comprehensive but streamlined. In identifying sub-dimensions, we selected those that help to illustrate the broader domain. To clarify, individuals need not adopt all sub-dimension practices all of the time in order to engage in each broader LMM domain effectively.

**Table 2:** Indicators of effective research leadership.

**Domains**	**Sub-dimensions**
Leader interpersonal practices	- Communicates openly, clearly, and transparently, engendering trust - Offers support and care, creating mutual connection and respect - Provides encouragement and expresses enthusiasm for individual and collective goals - Recognizes contributions and gives credit where credit is due - Listens with empathy and to understand others' perspectives - Delivers feedback that is timely, clear, constructive, and actionable - Encourages others to provide their input and empowers people to share their feedback
Leader ethics and integrity	- Models the values and standards for responsible behavior in science - Prioritizes data integrity, transparency, and open, reproducible science - Speaks positively and proactively about ethics, compliance, and research integrity - Invites regular group discussion about research integrity, rigor, and compliance - Addresses misbehavior and mistreatment, and does not tolerate misconduct
Team dynamics and research environment	- Facilitates a shared understanding of expectations among the team - Inspires team members around a common sense of purpose and belonging - Cultivates and prioritizes member learning, growth, and development - Creates a culture of teamwork, information sharing, and mutual problem solving - Fosters multi-directional constructive feedback and encourages members to ask questions; voicing concerns without fear of ridicule or retaliation is the norm - Expects and supports conflict resolution among team members - Prioritizes team member cohesion, engagement, wellbeing, and work satisfaction

The domains of effective research leadership, as illustrated in Table 2, focus on creating a constructive environment as a bedrock for team member behavior, interactions, and work activities. Through interpersonal practices such as open communication, expression of support, and giving and receiving feedback, leaders establish an environment of mutual respect and a shared sense of purpose (Antes et al., 2024). Importantly, leaders create social and psychological conditions for accomplishing complex, innovative work through their words, actions, and modeling ethics and integrity (Brown and Trevino, 2006, 2014; Brown et al., 2005; Carmeli and Gittell, 2008; Carmeli et al., 2013; Carmeli and Spreitzer, 2009). In this setting, team members feel safe sharing input and voicing concerns with one another and the leader, and the team can navigate conflict and setbacks (Antes et al., 2019a; Mumford et al., 2000c; Wefald, 2022; Antes et al., 2024; Silva et al., 2024; Jung et al., 2003; Ye et al., 2019; Gemmill and Wilemon, 1994; Mumford et al., 2013; Chen et al., 2021). Moreover, issues of responsible decision-making, data integrity, and research rigor and compliance are all open for conversation and areas of mutual commitment and growth.

With a constructive work environment in place through leadership, research management focuses on day-to-day operational and organizational processes to coordinate and make progress on projects. Research management aims to ensure work is reliable, trustworthy and rigorous (Antes et al., 2019a). As shown in Table 3, effective research management involves project management, team coordination and goal alignment, team ethics and integrity practices, and team member wellbeing (Antes et al., 2019a, 2024; Howard Hughes Medical Institute, 2006). Project management requires identifying and allocating resources, defining timelines, and ensuring follow-through on project goals. Organizing people to share knowledge, apply expertise, and mutually problem solve is essential for scientific work. This requires regular meetings to coordinate, plan, and gauge progress as a team, and opportunities for two-way feedback between members and supervisors (Antes et al., 2019a, 2024). Regarding ethics and integrity, effective research managers ensure team members develop and implement procedures to adhere to rules and regulations. Central to research management is also ensuring appropriate research recording keeping and documentation, management of data, and fair distribution of credit (Antes et al., 2019a). In complex, creative types of work, managers should monitor team member wellbeing, as a reasonable level of mental and emotional wellbeing is needed to perform detail-oriented work and engage in critical thought (Cai et al., 2018; Paterson et al., 2014).

**Table 3:** Indicators of effective research management.

**Domains**	**Sub-dimensions**
Project management	- Identifies and secures resources needed for projects - Allocates and monitors funds, tools, equipment, and people to accomplish the work - Identifies and oversees timelines, milestones, and schedules
Team coordination and goal alignment	- Facilitates knowledge sharing and utilization of expertise and team member strengths - Encourages collaborative decision - making and planning, or conveys reasoning when decisions need to be made unilaterally - Holds regular meetings to gauge progress and solve problems - Provides team members with constructive feedback in a timely and responsive manner
Team ethics and integrity	- Expects and oversees thorough research compliance, reporting, and documentation - Facilitates proactive discussion of authorship and intellectual property to adequately acknowledge others' contributions - Fosters open discussion about methods, data, and results - Expects responsible data sharing and management - Expects proactive disclosure of mistakes or concerns and responds constructively
Team member wellbeing	- Prioritizes high expectations and standards while also recognizing scientific work requires team members' mental and physical wellbeing - Expects and encourages members to find individualized approaches to maintain their wellbeing - Seeks solutions if members raise concerns about untenable workloads

Whereas leadership and management focus on the environment and team, effective research mentoring emphasizes the dyadic interaction between mentor and mentee. Mentoring adds domains found in Table 4, which are individualized to mentees. Effective mentoring includes understanding the unique needs and goals of individuals and providing individualized support and development tailored to this understanding (National Academies of Sciences Engineering and Medicine, 2019; Byars-Winston et al., 2018; Hinton, 2020). Likewise, in this dyadic relationship, the mentor models scientific values and integrity, shaping the mentee's awareness of research ethics and identity as a scientist (Plemmons et al., 2020; McGee et al., 2014; Wright et al., 2008). Finally, effective mentoring centers on aligned expectations, developing research competence and confidence in mentees, and guidance related to career advancement (National Academies of Sciences Engineering and Medicine, 2019; Masters and Kreeger, 2017). At its core, excellent mentoring entails individualized feedback and development of mentees in alignment with their needs and career goals (Pfund et al., 2016).

**Table 4:** Indicators of effective research mentoring.

**Domains**	**Sub-dimensions**
Individualized consideration and feedback	- Listens to individuals and develops an understanding of mentee goals and needs - Provides constructive career and professional development opportunities that align with the mentee's goals and supports their engagement - Offers guidance, training, and development for mentees with different goals, personalities, strengths, and challenges - Provides timely and constructive feedback on mentee research, career, and professional development - Recognizes that mentees' backgrounds and personal experiences may influence their experience in research and is willing to converse and explore these topics
Individual growth in ethics and integrity	-Conveys scientific values, norms, and standards in conversation and practices - Develops mentee understanding of the importance of research ethics and integrity in science - Models scientific integrity to mentees - Helps shape mentee professional identity as a scientist
Individual research competence and confidence	- Stimulates skill development and builds mentee confidence - Produces quality, rigorous research with mentees - Fosters independence and career advancement among mentees

We have noted key aspects of effective LMM, but LMM is not a one-size-fits-all approach for several reasons. First, context matters to a certain extent in the performance of LMM (Berson et al., 2001; Shalley and Gilson, 2004; Carmeli and Waldman, 2010; Oc, 2018). What constitutes effective LMM of a research lab at an undergraduate, liberal arts college may not mirror what constitutes effective LMM at a doctoral-granting academic medical center. Relatedly, LMM must consider the individuals involved, as the expectations, needs, skills, and experience of different trainees and staff can vary widely. Moreover, each person in a LMM role is unique, with their own personality and style, creating many potential approaches to effectively leading. While these contextual factors shape how individuals engage in LMM, context does not change the core tenets of effective LMM.

### LMM challenges

There are many challenges to performing LMM roles well. For instance, how much to direct team members vs. allowing them to self-direct their work is an important consideration in LMM. Another consideration is when to let a team member fail when failing might be important to their learning, but allowing failure may set back a project timeline. LMM requires setting expectations that are high yet realistic and establishing reasonable workloads. Likewise, how flexible to be with team members when objectives are not achieved, or when deadlines are missed, is another common concern of leaders and managers. Relatedly, handling when personal life events occur that derail work progress is a balancing act between compassion and productivity that leaders may experience as an ethical dilemma (Resnik et al., 2023).

Generally, it can be challenging to balance the needs, interests, and goals of team members with the needs, interests, and goals of the PI and their research agenda. As an example, balancing kindness and compassion while also holding high standards for work productivity can feel in tension initially (Sapienza, 2004, 2005). However, it is not necessary to be inconsiderate or heavy-handed to achieve these goals (Antes and DuBois, 2018). LMM based on mutual respect and growth often pushes others to succeed more effectively than anxiety and fear. Relatedly, engaging with each team member in an individualized manner can be challenging, but this approach is necessary (Dansereau et al., 1995; Okçu, 2014). It takes time to get to know people well enough to identify the nuances in their needs, and it takes attention and energy to be responsive to different people. One especially perplexing issue for some in LMM roles is when team members appear to have variable motivation. Figuring out what motivates different people to boost performance will be necessary, yet many PIs incorrectly expect that the discovery process itself will be the primary motivator for everyone (Al Rahbi et al., 2017; Antelo et al., 2010).

Perhaps most challenging is that there is limited time, or at least the perception of limited time, to engage in LMM activities. The link of LMM behaviors and practices to quality research, team dynamics, and research environments is not always understood. Thus, taking the time to engage in LMM can feel like a waste of limited time, or perhaps a luxury only for those who have already established themselves in their research fields (Greene et al., 2024). We argue that this is not the case; effective upfront approaches to LMM prevent downstream problems that waste time, such as interpersonal conflicts, staff turnover, difficulty attracting graduate students, mistakes in research, or even in the worst-case, research misconduct (McIntosh et al., 2020a; Galland et al., 2008).

Similarly, researchers may lack motivation or confidence to develop and apply the interpersonal skills necessary for effective LMM (Cameron et al., 2013). Some may believe that these practices are not worth pursuing unless done perfectly or effortlessly. However, it is essential to remember that striving for perfection should not hinder progress and the pursuit of good. To build confidence in these skills, researchers must begin practicing them in real-world settings (Hargie, 2021). As these skills are refined over time, researchers are likely to become more confident and comfortable in their application. Reflecting on what works well and what does not further enhances this learning process. However, this journey can be challenging, as researchers may be significantly influenced by their past experiences, which could include negative encounters with research mentors and leaders.

### What to measure to evaluate LMM effectiveness

Evaluation in any context begins with identifying what exactly it is that will be measured. What is selected to be measured must be clearly defined and have a reliable, valid measurement tool to assess it. We also recommend including someone with expertise in measurement and evaluation (e.g., a psychometrician, experts in the social sciences, workplace psychologists) on the team that develops and deploys an LMM evaluation plan.

We explore what institutions might consider measuring to understand LMM effectiveness in research teams or laboratories. In general, using a variety of sources of measures (e.g., PI, team members, supervisors) and a variety of types of measures (e.g., objective, subjective) offers a more robust understanding of the functioning of a team and those leading, managing, and mentoring the team. Everything that is measured ideally must also be actionable. If the information about a person's LMM performance cannot be intervened on, then the measure's utility is limited.

One general distinction in measures might be if they are “hard” objective metrics that are quantifiable and able to be observed directly vs. “soft” subjective metrics that involve people self-reporting their attitudes, perspectives, or experiences. Likewise, some measures might be collected at the individual level (e.g., only the PI completes the measure) or at the team level (e.g., all team members complete the measure). Team-level metrics are often aggregates of all individual team member responses. Given significant overlap in LMM functions and outcomes, and that all are necessary for research team success, we encourage using a set of metrics that examine a combination of subjective and objective factors at the individual and team level. It is possible to gather objective measures of LMM practices and outcomes, such as whether and how many regular formal or informal meetings occur, whether formal training is provided, the presence of a lab manual or scientific protocols in the lab, and turnover of staff.

In Table 5, we present example measures relevant to LMM, but these measures do not reflect an exhaustive list. Most measures included assess LMM and related practices from the perspective of mentees or team members. It should also be noted that the LMM measures include both process and outcome measures. LMM processes can be thought of as LMM behaviors and practices (i.e., application of skills), whereas LMM outcomes can be thought of as LMM performance (i.e., effectiveness and impact).

**Table 5:** Measures for assessing LMM in research.

**Domain**	**Description**	**Potential metrics to measure**	**Example measures**
Leadership	Measures assess the style, behaviors, practices, skills, attitudes, effectiveness, or impact of the leader.	- Clarity of communication about expectations - Ethical leadership - Supportiveness - Self-awareness - Communication style - Emotional intelligence - Social skills - Leadership self-efficacy	- Leadership and Management in Science (LAMPS) (Antes et al., 2024) - Leadership Self-Efficacy and Implicit Theories (Burnette et al., 2010) - Self-awareness (Ashley, 2012)
Management	Measures assess the style, behaviors, practices, skills, attitudes, effectiveness, or impact of the manager.	- Meeting effectiveness - Role clarity - Communication style - Constructive guidance and feedback	- Leadership and Management in Science (LAMPS) (Antes et al., 2024) - Management Communication Styles Scale (Rozilah et al., 2013)
Mentoring	Measures assess the style, behaviors, practices, skills, attitudes, effectiveness, or impact of the mentor.	- Quality of relationship - Perceived mentor effectiveness - Research competence	- Mentor Effectiveness Scale (Byars-Winston et al., 2015) - Advisory Working Alliance Inventory (AWAI) (Schlosser et al., 2011)
Team environment or climate	Measures assess shared perceptions of policies, practices, and behaviors in a team.	- Climate for ethics - Interpersonal climate - Psychological safety	- Lab Climate for Research Ethics (Solomon et al., 2021) - Climate of Accountability and Interpersonal Respect (Martinson et al., 2023)^*^ - Edmondson's Psychological Safety Scale (Edmondson, 1999)
Team dynamics	Measures assess various aspects of how a team functions, interacts, or collaborates.	- Team cohesion - Team communication - Conflict resolution - Team practices	- Research Team Practices Measure (Antes et al., 2024) - Team Communication Behaviors Scale (Hartner-Tiefenthaler et al., 2022) - Team Satisfaction (Tekleab et al., 2009)
Team member attitudes	Measures assess team member attitudes influenced by LMM.	- Job satisfaction - Job engagement - Sense of belonging	- Brief Index of Affective Job Satisfaction (Thompson and Phua, 2012) - Belonging Scale (Blau et al., 2023) - Work Group Inclusion Scale (Chung et al., 2020)

A full review of mentoring measures is available in a report from the National Academies (National Academies of Sciences Engineering and Medicine, 2019). There are many team-level measures provided by the Penn State Clinical and Translatonal Science Institute in their online toolkit (PennState Clinical and Translational Science Institute, 2025). ^*^This measure is in preprint form only and not a peer-reviewed publication.

Ideally, when selecting measures to evaluate LMM in practice, the measures are adapted to be context-specific; this relevance increases face validity and engagement. In general, there are a limited number of LMM measures tailored specifically to academic contexts. In selecting example measures, we considered measures that were developed specifically for academic research or scientific domains and provided examples of these measures when possible. Especially for leadership and management, there are few measures available. Many mentoring measures designed for the research context are available. When selecting broader measures not specific to the academic research context, we reviewed potential measures to ensure they had some evidence of validity and identified whether the items appeared plausible for application in the academic research context.

As we conclude the discussion of potential measures to capture LMM in research, we should take a moment to note traditional measures of academic productivity and success. Typical academic performance metrics focus primarily on academic output, including the number of peer-reviewed publications, amount of external funding, conference presentations, invited talks, and similar metrics. Like all metrics, even these measures, which may seem relatively objective since they can be quantified, are not without limitations (Jones and Froom, 1994; Lim et al., 2025; Rice et al., 2020; Buller, 2012). For example, counts of manuscripts do not capture the quality or rigor of the work, so other measures like H-index have been devised to try to measure academic impact. Additionally, different research institutions may have different cultures and values, which means the importance of different metrics may vary by institution. For instance, when assessing faculty, some institutions put greater emphasis on teaching evaluations provided by students, while others place less emphasis on teaching. Being an engaged organizational citizen and participating in service activities to the department, institution, or discipline are also valued to varied degrees at different institutions.

While conventional academic metrics are not necessarily inappropriate, they are incomplete and limited in capturing the full scope of research leadership duties and responsibilities. Traditional measures focus on the outputs of research LMM without assessing the processes involved in achieving these outcomes. This oversight includes LMM behaviors and practices that significantly impact research environments, team member retention, learning, and wellbeing.

## Approaches for evaluating LMM effectiveness

There are multiple, complementary avenues for evaluating the effectiveness of LMM. We elaborate on three distinct methods: (1) top-down evaluation, (2) bottom-up evaluation, and (3) self-evaluation. Each method has its strengths and limitations, which is why leveraging all three approaches simultaneously is needed to balance the limitations of each. That is, not one method is necessarily better or worse than the other. This multi-pronged approach is akin to 360-degree feedback where insights are gathered from multiple sources and individuals (Kim et al., 2016; Smither et al., 2005). Generally speaking, multiple sources of evaluation and multiple metrics from different domains are ideal.

### Top-down evaluation

Top-down evaluation can be thought of as a more conventional means of assessing performance whereby evaluation takes place “from the top”, such as when a supervisor evaluates an employee. For example, performance reviews delivered by a department chair or someone in a similar role are an example of top-down evaluation. A key component of effective top-down evaluation begins with setting and communicating clear expectations about what constitutes quality LMM performance, that engaging in these behaviors is an important and expected part of the job, that they will be evaluated on their performance in these areas, and that these behaviors are connected to what the institution values. That means quality performance within these domains needs to be clearly defined and operationalized, as illustrated in the section about indicators of LMM effectiveness. To the extent possible, objective and observable indicators of quality LMM performance should be used.

One way to achieve objectivity is by using behaviorally anchored rating scales (BARS) that provide a clear standard for performance at different levels that is tied to specific observable behavioral markers. That is, BARS help reduce bias in assessing performance in multiple ways, including: using clearly defined and observable job-related behaviors that serve as benchmarks for assessing performance rather than using subjective opinions about someone's personal characteristics; providing standardized descriptions about different levels of performance that everyone uses as a reference point when completing evaluations, which reduces ambiguity and makes it easier for those performing evaluations to match observed job-related behaviors with the appropriate level of the rating scale; and mitigating the influence of general overall impressions (i.e., either positive or negative) about a person on assessing different facets of their job performance (Smith and Kendall, 1963; Pulakos, 1984).

To create a BARS, first determine the key LMM behaviors and skills of interest. For example, a key leadership practice may be prioritizing and cultivating respectful relationships with members of the research team. Then, identify through past critical incidents (e.g., through interviews, surveys, or direct observations of behavior), examples of both effective and ineffective performance of these practices. After that, establish a Likert rating scale [e.g., a scale of 1 (unacceptable) to 5 (outstanding)] that includes a behavioral definition for each point on that scale. Likert scales have historically been used in employment settings to provide a mechanism for measuring job performance in a structured and quantifiable way, including performance related to LMM (Jebb et al., 2021). Multiple Likert scale items can be combined to measure a single complex construct, such as leadership.

As an example of Likert scale definitions, a 5 for the leadership behavior prioritizing and cultivating respectful relationships might be defined as “consistently provides opportunities for lab members to provide feedback”, a 4 might be “usually provides opportunities for lab members to provide feedback”, a 3 might be “provides adequate opportunities for lab members to provide feedback”, a 2 might be “regularly requires prompting to provide opportunities for lab members to provide feedback”, and a 1 might be “rarely provides opportunities for lab members to provide feedback”. The specific wording of Likert scale anchors will depend on the construct being measured, but the general principle and approach to Likert scale development cuts across constructs in that each point in the scale quantifies job performance for a given domain. We also recommend validating the BARS by asking exemplar leaders, managers, and mentors to review scale definitions to verify that the definitions used are appropriate indicators of leadership, management, and mentoring performance. Adjust the BARS as necessary based on feedback provided.

Another key component to providing quality top-down evaluation is having routine check-ins about performance that involve delivering feedback on LMM performance—that is, top-down evaluation should include areas for growth. Put differently, annual performance reviews should not be the only time leaders, managers, and mentors are evaluated on and receive feedback about their performance from their bosses (Pulakos et al., 2015; Cappelli and Tavis, 2016). Those delivering feedback should reference clear examples of the individual's past LMM behavior and the impact of those behaviors rather than providing commentary that is overly general or personality focused. For example, instead of providing the feedback “your lab members don't think you are a good leader”, providing the feedback “some of your lab members have expressed frustration that you do not meet with them regularly or address their feedback” is less personal and more directive and actionable.

Feedback conversations should also be bidirectional, and both parties should be given the opportunity to share their perspective and ask questions (Ashford and Tsui, 1991; Huang et al., 2017; Meinecke et al., 2017). Consider beginning feedback conversations by asking the person who will be receiving feedback to share their views about how they feel things are going. This approach to starting the conversation will convey that the person delivering feedback cares about the feedback recipient's perspective and experience, making it more likely that they will positively receive the feedback that is about to be shared. Quarterly check-ins can help avoid delays with identifying and correcting performance issues. That is, feedback should be given as close to the relevant observed performance or behaviors as possible. Having performance reviews only annually also runs the risk of recency bias, where the person evaluating the performance tends to focus more on recent behaviors rather than behavioral patterns over the course of the year (Steiner and Rain, 1989). Providing more routine performance check-ins can also help balance focusing on past performance with focusing on future professional growth and development (Gnepp et al., 2020).

In the context of a faculty member being evaluated by a department chair, it may be rare for the department chair to have regular opportunities to observe the faculty member's LMM behaviors. Alternate sources of top-down evaluation may be senior or peer mentors, as these individuals are likely to have more opportunities for observing LMM behaviors compared to a department chair. In what follows, we talk about two other approaches to evaluation: bottom-up evaluation and self-evaluation. These two approaches are viable supplements to the top-down evaluation approach described here and make up for the limitations of this approach.

### Bottom-up evaluation

Bottom-up evaluation take place “from the bottom” in which research leaders are evaluated by their team members. Often, bottom-up involves bidirectional feedback between research team leaders and team members, so the flow of feedback goes both ways with the aim of improving collective team functioning. Of course, research team leaders must provide routine feedback related to projects, research progress, and other scientific activities. For the purposes of this paper, we focus specifically on the dynamic of the research leader soliciting, receiving, and responding to feedback on their LMM performance from their research team members. This process may include areas that the LMM and team members need to collectively grow and improve in their approaches to communication or collaboration.

In terms of bottom-up feedback that focuses on the leader receiving feedback from team members, this approach may be adopted independently by some team leaders but generally is not the norm. There are several factors to attend to for this approach to performance evaluation to be successful. First, research leaders must be willing to truly hear constructive feedback from lab members and not overreact, lash out, or retaliate in response to uncomfortable feelings that receiving this feedback may cause. This mindset requires humility, which is essential in high-stakes research environments because researchers are fallible human beings just like everyone else (Owens et al., 2013; Nielsen et al., 2009). By adopting a growth or a learning mindset, recognizing that there are always opportunities to improve and that constructive feedback is not a critique of the leader as a person, leaders can leverage novel perspectives and insights from team members to identify and act on areas for growth (Dweck, 2016).

A second consideration is that the research leader must proactively and consistently make a good-faith effort to create a psychologically safe environment where lab members feel comfortable speaking up and sharing constructive feedback about the leader without fear of being retaliated against (Edmondson and Lei, 2014; Van Noorden, 2018; Jin and Peng, 2024). Being on the receiving end of unwarranted negative reactions from a leader is a legitimate fear some lab members may have, even if the leader does not consider themselves to be a punitive person. There may also be cultural factors that affect lab members' willingness to give leaders feedback and the type of feedback shared with leaders about their performance (Hofstede, 1984, 2001). For example, in some cultures, directly criticizing a leader, even if constructive, could be viewed as disrespectful or inappropriate. To assuage possible fears, leaders should explicitly state to lab members that their candid feedback is important, valued, and welcomed. It may also help to articulate why their feedback matters and plans for how that feedback will be used. For example, the lab leader could say that both positive and constructive feedback are important for the lab leader's growth, the success of the lab, and the quality of the work environment and the scientific work itself.

Like top-down evaluation, bottom-up evaluation should ideally take place periodically (e.g., quarterly) rather than once annually. This does not necessarily mean that a formal bottom-up evaluation must take place during multiple pre-planned instances per year; solicitation of feedback can also be done informally or on a rolling basis. Lab leaders could consider making available avenues for providing anonymous feedback when possible, as doing so may facilitate sharing of more candid feedback compared to if identifiable feedback was solicited. If a lab or research team is small (e.g., 5 members or less), this anonymized approach may not be feasible. However, for larger teams, online surveys can be used to collect lab member feedback anonymously.

Feedback surveys should include both Likert scale and open-ended questions mapped to the LMM dimensions of interest. Examples of Likert scale questions might involve asking lab members to rate on a scale of 1 (strongly disagree) to 7 (strongly agree) a series of statements, such as “*The lab leader effectively involves the research team when making decision*s” or “*The lab leader clearly communicates expectations for performance on research projects*.” Examples of open-ended questions might be, “*What changes could the lab leader implement to improve the team dynamics in the lab?*” or “*What are specific ways the lab leader can better support your professional development?*”. Survey data can be aggregated and analyzed to identify patterns of feedback from lab members as a collective.

Leaders should consider preparing their lab members to give feedback by providing them with guidance or training on how to provide useful and actionable feedback (e.g., delivering feedback in a respectful manner, engaging in empathy and perspective taking, framing feedback in a manner focused on growth and suggestions for realistic future changes rather than only pointing out shortcomings, focusing on specific behaviors). It is likely that lab members have limited understanding of the leader's responsibilities and are unprepared to effectively evaluate someone's performance. Providing training can also help support the quality of feedback shared by lab members to avoid feedback that is overly general, vague, or emotionally based (Rosales Sánchez et al., 2019; Roch et al., 2012).

A final consideration is that, for bottom-up evaluation to be successful, leaders need to be willing to make tangible changes in how they lead, manage, and mentor, and actually respond to or follow through on the feedback they receive from lab members. Asking for feedback and not acting on it is just as bad as or worse than not soliciting feedback at all (Milliken et al., 2003; Detert and Burris, 2007; Robinson and Morrison, 2000; Ashford and Cummings, 1983). Some feedback may not be easy, quick, or feasible to implement, so leaders must share (e.g., during a lab meeting) the rationale for why some feedback may not be implemented as readily. However, for feedback that is actionable, leaders can share with the lab concrete plans for what specific changes are being made in response to feedback provided and over what timeframe. Additionally, lab leaders may want to gather input on the feedback process and make adjustments based on what is and is not working well for their team. There are multiple ways to solicit and implement feedback effectively.

### Self-evaluation

Self-evaluation is a mechanism for increasing self-awareness, which is an essential part of personal and professional growth and satisfaction. Self-evaluation involves engaging in self-driven, purposeful information gathering, reflection, planning, and intention setting. These processes work in tandem to help leaders gain insights into themselves and their LMM performance, identify areas for improvement and change, and create realistic plans for implementing change that are in alignment with their goals (Anseel et al., 2009). Self-evaluation should be a standard part of research leaders' professional routines.

Reflection, a key component of self-evaluation, is the exploration of one's thoughts, behaviors, and emotions to help a person come to a better understanding of themselves. Consistently and periodically dedicating time and space for reflection will help ensure self-reflection actually happens and that the benefits of self-reflection are maximized. Especially with demanding research careers, leaders of research teams may feel that spending time on self-reflection is frivolous or not a worthwhile use of their limited time. However, self-reflection is an important part of the job, as doing so ultimately has direct implications for multiple aspects of their scientific work and the teams they lead, manage, and mentor.

When engaging in reflection, leaders should focus on both their accomplishments (e.g., what was done well) and areas for growth (e.g., what could be done differently) in all LMM domains. Leaders can keep a running list of accomplishments to refer to as a way of measuring progress. Examples of personal life accomplishments may include starting a family, having time to spend with loved ones and friends, engaging in hobbies, and traveling. Examples of professional accomplishments may include grants, publications, trainee success stories, project success stories, positive feedback from trainees and colleagues, and awards. When reflecting on areas for growth, leaders can identify both challenges and mistakes. Much like an after-action review (Crowe et al., 2017), reflecting on challenges or mistakes can help leaders understand and learn from the root causes of these issues and how those issues might be prevented in the future.

To boost the effects of reflection, leaders can articulate and reflect on short-, mid-, and long-term personal and professional goals, as goals can be linked with areas for growth. Goals should be clear, specific, measurable, observable, and behaviorally-oriented (Locke and Latham, 2002; Gollwitzer and Sheeran, 2006). For example, a leadership-focused goal might be to hold an annual lab event where lab members are recognized for their work and contributions. A management-focused goal might be to provide a lab-wide refresher training on a project's protocol each year. A mentoring-focused goal might be to spend a dedicated portion of one-on-one meetings with lab members listening to them about their career goals. Each leader will craft unique goals and should be candid with themselves about what goals are realistic, given their life and career circumstances.

Reflection is a necessary but insufficient step toward quality self-evaluation. Leaders must be able to translate this reflection into action and behavior change. One way to do this is by creating and implementing an action plan. Action plans are a roadmap that specifies what it is that leaders want to change, over what timeframe, what resources and information are needed, and who can help hold the leader accountable for following through on this plan (Gollwitzer and Sheeran, 2006; Gollwitzer, 1999, 1997; Orbell et al., 1997). Part of action plan implementation involves monitoring progress on the plan. Various approaches can be taken to monitor progress, including journaling, using performance tracking software, using Excel spreadsheets, and soliciting input on progress from peers, mentors, coaches, and lab members.

It is also important to be aware of the limitations of self-evaluation. Self-evaluation is subject to self-serving biases, where leaders downplay LMM mistakes or errors and embellish or exhibit overconfidence in LMM successes and skills (Campbell and Sedikides, 1999; Ross, 1977; Oswald et al., 2004). They may selectively attend to information that confirms existing beliefs they have about themselves or disregard constructive feedback that challenges their self-perception. In contrast, others may overemphasize shortcomings and underplay accomplishments and strengths. In both cases, leaders should be aware of challenges with identifying their blind spots and prioritize adopting a balanced approach to evaluating themselves. Another limitation to self-evaluation is the risk of undue influence of emotions (Ilgen and Davis, 2000; Belschak and Den Hartog, 2009). For example, recent positive or negative feedback may leave leaders in an emotional state that skews their self-assessment. Leaders should consider engaging in self-evaluation at a time when their emotions are at a healthy, neutral, and objective baseline. To help manage emotions, leaders will likely need to practice emotional regulation and mindfulness. Other approaches to evaluation (i.e., top-down, bottom-up) can help offset various limitations of self-evaluation.

## Supporting evaluation and application of LMM in real-world settings

Meaningful institutional support is needed to successfully implement and fully reap the benefits of the various strategies and approaches described in this paper. Other institution-level mechanisms and practices are needed to reinforce effective evaluation and implementation of LMM practices. In what follows, we highlight key considerations for institutions and their leaders as they seek to support LMM.

### Communicating senior leadership's expectations about LMM

Senior leaders at academic institutions (e.g., presidents/chancellors, vice presidents, provosts, deans, associate deans, department chairs) must first be collectively committed to LMM and then explicitly articulate to personnel at their institution that quality LMM is valued and expected. Stated expectations should also include good-faith involvement in providing top-down and bottom-up evaluations for others when appropriate. As these expectations are communicated, institutional leaders should describe how quality LMM aligns with the institution's mission, vision, and values. Institutional leaders must also lead by example through modeling the LMM practices they expect from others at their institution. This includes soliciting and meaningfully responding to bottom-up and top-down (or peer) evaluations and engaging in self-evaluations themselves. Doing so will help set and convey the standard for others to follow. Leaders who do not engage in the LMM behaviors they expect from others will likely undermine attempts to cultivate quality LMM throughout their institution.

### Framing evaluation of LMM as professional development

It is not uncommon for individuals to experience evaluation apprehension, or anxieties or fears about being evaluated, especially if performance evaluation is done with punitive framing. These anxieties may be exacerbated when performance is being evaluated by peers or subordinates, which are historically uncommon sources of performance evaluation in academic settings. Evaluation apprehension can stymie transparent communication and lead to defensiveness, neither of which are conditions for effective performance evaluation. Framing LMM evaluation as a professional development tool can help mitigate evaluation apprehension and cultivate a growth mindset that helps people feel empowered to make positive changes in how they lead, manage, and mentor others (London and Smither, 2002). Similarly, articulating how LMM can help individuals achieve their professional goals and success can help motivate and cultivate buy-in from others. Giving training to those providing top-down, peer, or bottom-up evaluations of others can improve the accuracy of their evaluations and reduce subjectivity in their judgments (Rosales Sánchez et al., 2019; Roch et al., 2012). As a result, fears and anxieties about receiving biased reviews from others may be reduced.

### Providing dedicated time and support for LMM

Simply put, learning about LMM and effectively engaging in LMM practices takes time, effort, and resources. It also takes time to see the effects of LMM on the scientific work being done and the research environment. These considerations should be taken into account as expectations are set about individual workloads and performance in other domains of a person's job (e.g., publishing, grant writing) (Mazzetti and Schaufeli, 2022; Spreitzer and Quinn, 1996). Providing protected time to learn about and engage in LMM practices will help support their adoption. Similarly, encouraging and providing dedicated time for self-reflection as a means of self-evaluation is important, as people may be reluctant to routinely engage in reflection because they do not think it is job-relevant or a good use of their time.

Investing in formal LMM training programs for individuals across career stages and roles is an additional strategy institutional leaders can leverage to cultivate LMM institution-wide (Van Noorden, 2018). Scientists are often taught how to do science within their area of subject matter expertise but have historically not been taught how to lead, manage, and mentor others which requires an entirely different skillset. While it is important for core LMM principles to be taught across training programs so that there is a thread of continuity, the specific content and approach for each program will likely need to be tailored depending on the group of interest. For example, a LMM training program for early-career researchers will likely emphasize different points compared to a LMM training program for senior faculty or staff. These programs can take various forms, including half-day or full-day workshops, semester-long synchronous or asynchronous courses, and micro-learning that involves delivering content in small (e.g., 15 min) segments that are focused on a specific topic or objective.

### Establishing appropriate incentive structures for LMM

Employee motivation is complex, but establishing appropriate incentive structures can help reinforce LMM practices and directly affect motivation, job satisfaction, and productivity (Kerr, 1975). Appropriate incentive structures are those that are linked to both intrinsic and extrinsic motivation. In terms of intrinsic motivation, people want to feel that they belong, work in an environment where they feel respected and needed, and are doing something meaningful or impactful with their time. Examples of incentives aligned with intrinsic motivation include being able to use their skills, working in an environment and for a boss and team that supports them, being given opportunities to learn new skills, being encouraged and recognized for their work, and having a voice about decisions that affect them. With extrinsic motivation, people want to be fairly compensated and receive recognition for their work. Examples of incentives aligned with extrinsic motivation include being given opportunities to advance in their career, being paid well and receiving consistent pay raises, being promoted, being given support to go to conferences or other career-related programs, and being given adequate or even extra time off of work to recharge. Incentive structures that tap into both intrinsic and extrinsic motivation can propel the adoption of effective LMM practices; that is, what gets rewarded and incentivized gets done.

### Institutional risk factors that may undermine LMM goals

In academic settings, there are likely practices, norms, and cultures currently in place that may, while enacted with good intentions or with the intent of upholding tradition, undermine attempts at cultivating effective LMM. One such practice is a near-exclusive emphasis on research output, namely the number and amount of publications and grants (Lim et al., 2025; Rice et al., 2020; Buller, 2012). This emphasis may show up in annual performance reviews and during tenure and promotion decisions. To be clear, it is not that these metrics are unimportant—they are undoubtedly a key part of a scientific research career—rather, these metrics should be considered as part of a larger menu of performance domains.

Asking employees, especially faculty, to shoulder excessive administrative duties or bureaucratic processes may unintentionally undermine LMM. While the concept of “service” is often considered during promotion and tenure decisions, and being a good institutional citizen is a professional responsibility, these requirements should not substantially detract from the time and energy needed for other more essential parts of the job, including the scientific work and LMM. In this same vein, asking others to provide peer, top-down, or bottom-up evaluations of others places a demand on their time. During busy evaluation periods, institutional leaders should consider adjusting workloads to meet these demands, as providing quality evaluation of others takes notable bandwidth.

Unfair or inequitable resource allocation may also undermine effective LMM. Policies that allocate resources based primarily on research or clinical dollars brought into the institution can hinder the LMM success of faculty whose work naturally brings in less money. Certain departments or centers may organically attract greater resources to provide support for and training on LMM to faculty, staff, and trainees. It is in the institution's best interest to ensure everyone receives equitable support for LMM. Similarly, having unclear or inconsistent institutional policies related to LMM may disrupt attempts to cultivate effective LMM institution-wide. While institutions are likely to have some relevant policies in writing, they may be written in such a manner that is overly vague or unclear. Lack of transparency about and consistency in applying policies can also erode trust that faculty, staff, and students have in the institution's administration. Finally, employing trainees as staff or faculty after their graduation without consideration of other qualified applicants or individuals who align with a narrow set of personalities and styles can undermine effective LMM. These practices can limit the extent that new LMM approaches and styles are assimilated into the organization, or worse, can perpetuate bad or harmful LMM practices.

To be effective in LMM, researchers must have adequate support for work-life balance. Societal norms about when and where people work have shifted in recent years, especially since the COVID-19 pandemic. Institutional policies that fail to consider issues related to work-life balance (e.g., the need to work remotely for a period of time to accommodate childcare needs; the need to schedule a medical appointment during the workday) may contribute to burnout or turnover intentions that put effective LMM at risk.

### Launching LMM evaluation institution-wide

Especially if institutions do not have a LMM evaluation system in place or do not have experience with evaluating LMM generally, institutional leaders may want to implement LMM evaluation in a stepwise fashion. For example, institutional leaders could initially pilot certain LMM evaluation approaches in a department or division to see what does and does not work well, prior to rolling out the evaluation institution-wide or to a larger group. It is also worth noting that implementing a new LMM evaluation system institution-wide can be disruptive and stressful. Setting up such a system well takes time, excellent communication, and careful planning and implementation; it should not be done haphazardly or as an afterthought. As LMM evaluation systems are ramped up, institutional leaders may also want to consider tracking LMM evaluation data over time to examine trends in LMM performance across the institution. Doing so can help identify areas where additional LMM support is needed within the institution.

## Conclusion

Researchers are taught how to conduct technical scientific work during their formal training, but it is increasingly becoming more critical for researchers to learn LMM skills. Just as there is a need to learn LMM skills, there is also an increased need for more robust institution-wide evaluation of and support for LMM skill development and application. There is not a one-size-fits all approach to LMM and LMM are multifaceted, which can make evaluation of LMM challenging. Yet, we have proposed a range of metrics at the individual and group levels that can capture key aspects of effective LMM and healthy research environments. We hope that the LMM evaluation framework and examples provided are helpful for institutional and lab leaders seeking to take the next step forward in LMM evaluation.
